# Salvage of buccal mucosa graft pyeloplasty in kidney transplantation: A case report

**DOI:** 10.1016/j.eucr.2025.103215

**Published:** 2025-09-15

**Authors:** O. Decombe, A. Manuguerra, A. Blondeau, C. Larose, A. Flahault, C. Mazeaud

**Affiliations:** a Department of Urology, CHRU Nancy, Université de Lorraine, Nancy, France; b Department of Nephrology and Kidney Transplantation, CHRU Nancy, Université de Lorraine, Nancy, France; c IADI-UL-INSERM (U1254), Nancy, France

**Keywords:** Buccal mucosa graft, Ureteral reconstruction, Kidney transplantation

## Abstract

We report the first successful use of a buccal mucosa graft for pyelo-ureteral fistula repair in a renal transplant recipient under immunosuppressive therapy.

A 73-year-old male developed a urinary fistula following kidney transplantation. Surgical revision revealed a 2-cm anterior wall defect, which was repaired using an *onlay* buccal mucosa graft and omental flap. Postoperative outcomes were favorable, with no leakage, stable renal function, and preserved graft perfusion.

This case highlights the feasibility and effectiveness of buccal mucosa grafting as a minimally morbid alternative to conventional techniques in complex ureteral reconstruction, even in immunosuppressed patients.

## 1 Introduction

Buccal mucosa grafting has become the leading procedure for the treatment of recurrent urethral strictures. Due to its biological properties, such as good vascularization and resistance to urine exposure, buccal mucosa graft emerged as a new alternative for native ureter repair. Using buccal mucosa for proximal, long, and complex ureteral reconstruction is efficient, with satisfying efficacy and no significant complications.^1^ This surgical technique avoids the morbidity associated with the interposition of the intestine or kidney auto transplantation, making management easy and safe for both the surgeon and the patient.^2^

## 2 Case

Mr. M., aged 73, underwent a kidney transplant in May 2024 in the context of chronic renal failure due to IgA nephropathy and has been on hemodialysis since 2021 with residual diuresis at 500 mL per day. His previous history included appendectomy, and exposure to asbestos, which is responsible for pulmonary hypertension and probable chronic obstructive pulmonary disease.

The kidney graft came from a cadaveric donor with a panel reactive antibody (PRA) of 0 %. The surgical procedure went as planned: we performed a renal graft of a left kidney in the right iliac fossa with modal vascular anastomoses on the external iliac vessels with one artery, one vein, and a pyeloureteral anastomosis with a double running suture of PDS 6.0, because the graft ureter appeared to have poor vascularization.

The patient received induction therapy with thymoglobulins, and then corticosteroid therapy was stopped early on day 7 after surgery. He received immunosuppressive treatment with MYCOPHENOLATE MOFETIL and TACROLIMUS. The functional outcome during the post-operative period was within normal limits. The recovery of renal function started early on postoperative day (POD) 2, with a diuresis of 35 mL/h and creatinine levels at 600 μmol/L, reaching a stable level of around 190 μmol/L at 1 month post-operatively. Doppler ultrasound of the graft, performed on POD 1, showed a good vascularization of the graft, without any pelvic dilatation, infiltration, or peri-transplant collection.

**Fig. 1:**
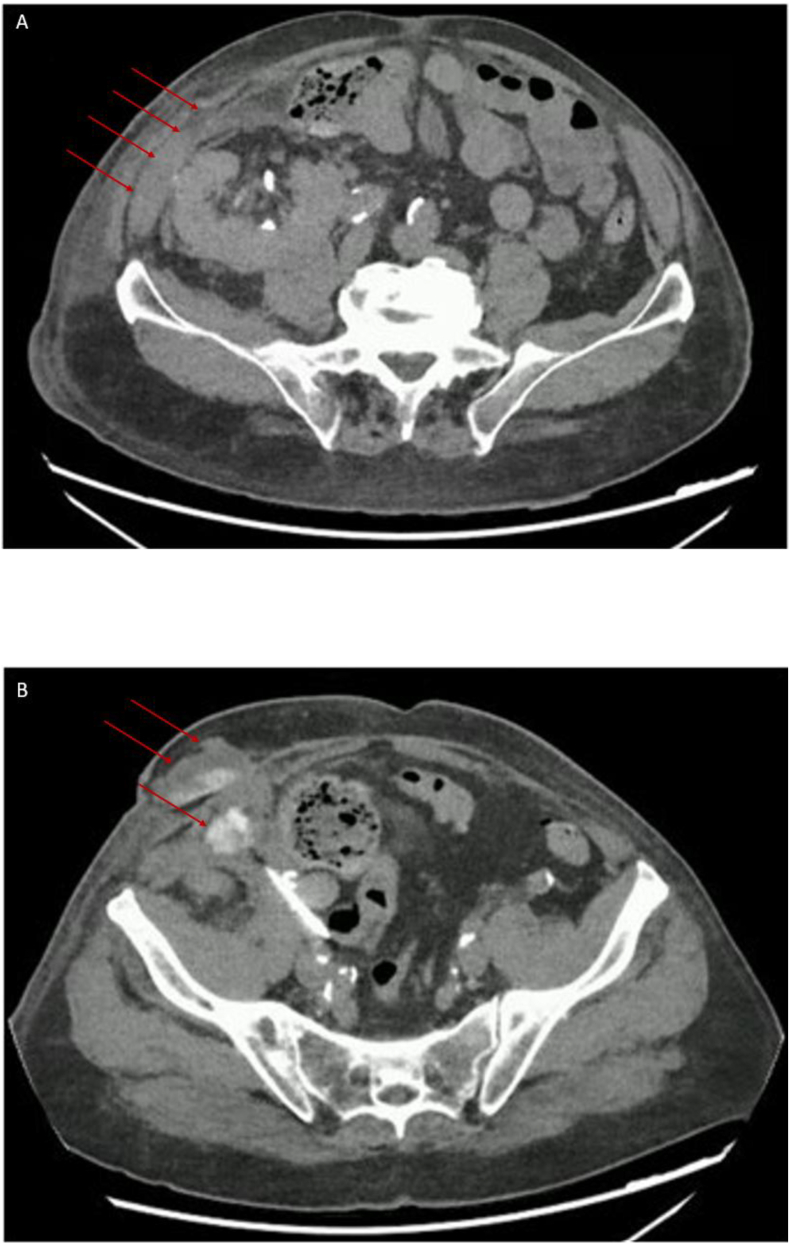
**Baseline CT-scan revealing a urinary fistula despite nephrostomy and monoJ-stent drainage** (A) Arrows demonstrate a urinoma on the anterolateral side of the renal graft; (B) Arrows demonstrate a urinoma extending into the anterior abdominal wall and forming a fistula through the surgical wound.

On POD 12, a urogram was performed due to serous discharge from the surgical wound. Fluid analysis revealed a creatinine level of 3700 μmol/L (compared to 200 μmol/L in serum), and imaging demonstrated a 9 cm fluid collection adjacent to the graft. The collection was extended to the subcutaneous tissue and showed contrast enhancement at 1 h, closely related to the proximal portion of the double-J stent, suggesting a urinoma. A mono-J stent was placed endoscopically to optimize pelvic drainage. Fig. 1.

On POD 24, persistent wound discharge was noted, with a daily output of 500 cc and elevated creatinine levels in the fluid. A nephrostomy was placed to complete the urinary drainage alongside the mono-J stent.

**Fig. 2:**
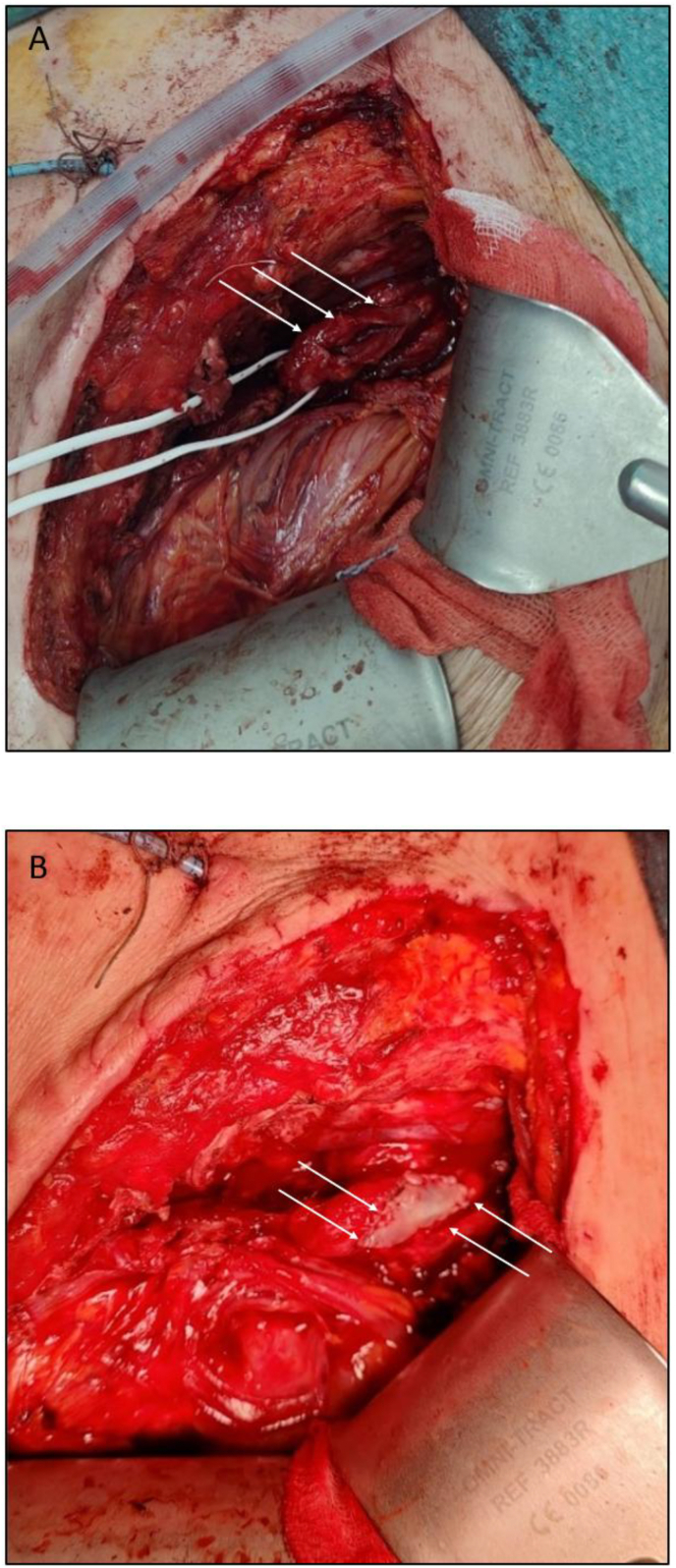
**Perioperative images** (A) Open pyeloureteral anastomosis on the anterior side (arrows), prior to buccal mucosa graft placement; (B) Buccal mucosa graft on the renal graft pelvis, proximal to the pyeloureteral anastomosis (arrows).

Therefore, at POD 28 we finally perform open revision surgery to repair the pyeloureteral anastomosis: a 2-cm anastomotic fistula with a longitudinal anterior opening was identified. The opposite side remained attached, forming a continuous and well-vascularized posterior wall. Thus, we performed a salvage pyeloplasty using a 2-cm autologous buccal mucosa graft under favorable conditions with *an onlay* technique, secured by a double running suture of PDS 6.0, and reinforced by an interposed omentum flap brought in through a window in the peritoneum. A new 7/24-cm double-J stent was placed to support healing, and the nephrostomy tube was left for safety. The nephrostomy was removed one month after the open revision surgery, following a clamping test. Fig. 2.

At 4 months, a follow-up CT-scan showed subtotal clearance of the peri-transplant collection. Furthermore, renal function remained stable (146 μmol/L).

The ureteral stent was removed 6 months after buccal mucosal graft pyeloplasty. A Doppler ultrasound performed the following day showed no pyelocaliceal dilatation.

**Fig. 3:**
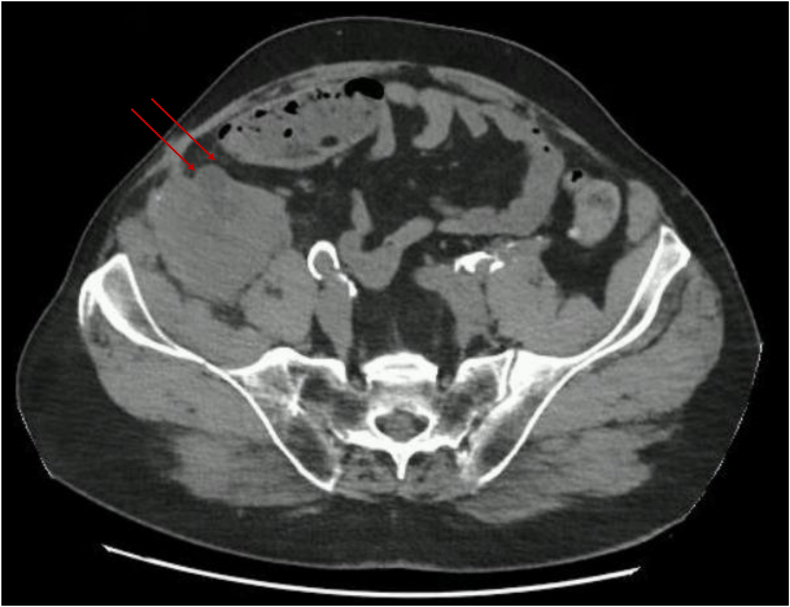
**CT-scan from the last follow-up (2 months after the double-J stent removal)** Renal graft pelvis (arrows), without pyelocaliceal dilatation or adjacent collection.

The long-term urological outcome was favorable. Two months after the ureteral stent removal, a follow-up abdominopelvic CT-scan was performed without contrast agent injection due to a GFR of 29 mL/min. This scan showed complete clearance of the collections around the graft and no dilatation of the graft's pyelocaliceal cavities. Fig. 3.

One year after the buccal mucosa transplant, a follow-up ultrasound revealed an 11 cm kidney transplant with good corticomedullary differentiation and no pyelocaliceal dilatation (10 mm), with no collections around the graft. Additionally, kidney function remained stable.

## 3 Discussion

Urinary stenosis and fistulas are well-known complications of uretero-vesical and pyeloureteral anastomoses in kidney transplantation. While less common than anastomotic strictures, urinary fistulas remain clinically significant. For instance, *Santos and al.* reported a 2.16 % incidence of fistulae versus 3.24 % of strictures in a cohort of 648 renal transplants. Primary treatment was open surgical revision in 52 % stenosis and 100 % fistulas, with a success rate of 71 % for the fistulas. Three patients required surgery as a secondary approach with 100 % success.^3^

We report the first case of urinary fistula management post-renal transplantation using buccal mucosa graft under immunosuppressive therapy. Following close postoperative monitoring, this innovative approach yielded promising outcomes. Buccal mucosa grafts are an established option in ureteral stricture repair, with a pooled complication rate of 9 % beyond three months, according to a meta-analysis by *You* et al.*.*^2^ However, their use in immunosuppressed transplant recipients has not previously been described.

Duty et al. conducted a review of several studies on post-operative complications after kidney transplantation. Their analysis reported an incidence of urinary fistula between 1.2 % and 8.9 %, with the primary risk factor being devascularization of the transplant ureter during organ harvest. Initial management, as recommended in their report, includes placing a nephrostomy tube and a Foley catheter to improve urinary drainage. They found a success rate of 62 % (range: 36–87 %) after an average follow-up of 35 months. In our case, the ureteral fistula could not be effectively managed with conservative measures, despite using a low-pressure drainage system, and required surgical revision as suggested in this review. The initial pyeloureteral anastomosis limited surgical options. A pyelovesical anastomosis was considered high-risk because it involves creating a bladder flap, which carries inherent risks such as anastomotic stenosis and difficult bladder mobilization, especially one month after transplant.

As the pyeloureteral fistula was located on the anterior wall of the renal graft's pelvis, reconstruction using an *onlay* buccal mucosa graft was considered the most suitable option, preserving the posterior plane—crucial for vascularization, healing, and prevention of stenosis.^4^

Additionally, an omental flap was interposed through a limited pelvic peritoneal window to improve local angiogenesis, provide mechanical protection, and decrease the risk of fistula recurrence. One year after surgery, the outcome was favorable, and the patient remained stent-free despite ongoing immunosuppression. The histological compatibility of buccal mucosa with urothelial tissues likely contributed to the successful result.

Alternative treatments for persistent urinary fistulas include uretero-ileoplasty and transplantectomy. While effective, uretero-ileoplasty is more invasive and associated with higher morbidity.^5^ Transplantectomy remains a last-resort option when the graft is non-functional or responsible for significant complications.

## 4 Conclusion

The anastomotic fistula healed with a salvage graft of buccal mucosa attached to the anterior face of the pyeloureteral anastomosis. At one year, the patient was stent-free, showed stable creatinine levels with no pelvic dilatation while on immunosuppressive drugs. Postoperative management of ureteral fistula associated with renal transplantation remains complex and requires case-by-case assessment.

## CRediT authorship contribution statement

**O. Decombe:** Writing – review & editing, Writing – original draft, Resources, Methodology, Investigation, Data curation, Conceptualization. **A. Manuguerra:** Resources, Project administration, Formal analysis, Data curation. **A. Blondeau:** Visualization, Validation. **C. Larose:** Visualization, Validation. **A. Flahault:** Writing – review & editing, Visualization, Validation, Project administration, Investigation, Data curation. **C. Mazeaud:** Writing – review & editing, Visualization, Validation, Project administration, Methodology, Investigation, Conceptualization.
